# Direct Pathway Neurons in the Mouse Ventral Striatum Are Active During Goal-Directed Action but Not Reward Consumption During Operant Conditioning

**DOI:** 10.3390/biomedicines12122755

**Published:** 2024-12-02

**Authors:** Stefano Cataldi, Clay Lacefield, N Shashaank, David Sulzer

**Affiliations:** 1 Department of Psychiatry, Division of Molecular Therapeutics, New York State Psychiatric Institute, Columbia University, New York, NY 10032, USA; stefano.cataldi@nyspi.columbia.edu (S.C.); clay.lacefield@nyspi.columbia.edu (C.L.); 2 Italian Academy, Columbia University, New York, NY 10027, USA; 3 Division of Systems Neuroscience, New York State Psychiatric Institute, New York, NY 10032, USA; 4 Departments of Computer Science, Shapiro Center for Engineering and Physical Science Research, Columbia University, New York, NY 10027, USA; shashaank.n@columbia.edu; 5 New York Genome Center, New York, NY 10013, USA; 6 Departments of Neurology, Columbia University, New York, NY 10032, USA; 7 Departments of Pharmacology, Columbia University, New York, NY 10032, USA

**Keywords:** ventral striatum, operant conditioning, spiny projection neurons

## Abstract

Background/Objectives: Learning is classically modeled to consist of an acquisition period followed by a mastery period when the skill no longer requires conscious control and becomes automatic. Dopamine neurons projecting to the ventral striatum (VS) produce a teaching signal that shifts from responding to rewarding or aversive events to anticipating cues, thus facilitating learning. However, the role of the dopamine-receptive neurons in the ventral striatum, particularly in encoding decision-making processes, remains less understood. Methods: Here, we introduce an operant conditioning paradigm using open-source microcontrollers to train mice in three sequential learning phases. *Phase I* employs classical conditioning, associating a 5 s sound cue (CS) with a sucrose–water reward. In *Phase II*, the CS is replaced by a lever press as the requirement for reward delivery, marking an operant conditioning stage. *Phase III* combines these elements, requiring mice to press the lever during the CS to obtain the reward. We recorded calcium signals from direct pathway spiny projection neurons (dSPNs) in the VS throughout the three phases of training. Results: We find that dSPNs are specifically engaged when the mouse makes a decision to perform a reward-seeking action in response to a CS but are largely inactive during actions taken outside the CS. Conclusions: These findings suggest that direct pathway neurons in the VS contribute to decision-making in learned action–outcome associations, indicating a specialized role in initiating operant behaviors.

## 1. Introduction

The ventral striatum (VS) plays a pivotal role in reward processing, reinforcement learning, and operant conditioning. These processes are essential for adapting behavior to a constantly changing environment, as the brain maintains representations of where, when, and how to acquire components necessary for survival. These representations are built from experiences with sensory predictors in the environment and are continuously updated [1]. In neuroscience, the term reward refers to tangible reinforcers, such as food, psychostimulants, or electrical stimulation of ventral midbrain dopaminergic neurons, that drive action selection and goal-directed behaviors. This trajectory from sensory predictor to reward achievement is central to both adaptive and maladaptive behaviors, including addiction [2].

Operant conditioning is a process in which animals learn to associate behaviors with consequences, leading to an increased or decreased likelihood of repeating an action in the future. This form of learning is distinct from classical conditioning, where a neutral stimulus is paired with an unconditioned stimulus to elicit a response. In operant conditioning, behavior is driven by the pursuit of rewards or the avoidance of punishments [3]. Dysfunctional reward processing and maladaptive cue–reward associations are implicated in various disorders, including addiction and schizophrenia, where environmental cues can trigger pathological behaviors like drug-seeking or hallucinations [4,5,6]. Understanding how neural circuits in the VS process information during associative tasks can shed light on these conditions.

At the core of this reward-based learning in the VS are spiny projection neurons (SPNs), which form the majority of neurons in the striatum and are the neurons that project from the striatum to other nuclei in the basal ganglia and eventually to other regions of the nervous system. These neurons belong to two distinct pathways: direct pathway SPNs (dSPNs), which express D1 dopamine receptors, project to the globus pallidus interna and substantia nigra reticulata, and are understood to facilitate actions, and indirect pathway SPNs (iSPNs), which express D2 receptors, project to the globus pallidus externa, and are thought to inhibit actions [7,8,9,10]. The ventral striatum receives dense innervation from dopamine-producing neurons in the ventral tegmental area (VTA), which encode reward prediction error—shifting their activity from responding to the reward itself to responding to the associated cue over time [2,11,12]. While dopamine has long been linked to cue–reward associations, recent studies suggest that dSPNs in the VS play an active role in shaping the brain’s response to rewards, beyond being passive recipients of dopaminergic input [1,13].

In this study, we investigated the neural dynamics underlying cue–reward associations using in vivo imaging of calcium activity in dSPNs of mice performing an operant task. Mice were trained on a sequence of behavioral paradigms, progressing from classical to operant conditioning, to explore how dSPNs encode reward-predictive cues and motor behaviors. These findings provide insight into how the VS integrates sensory and motor information to guide behavior and could illuminate mechanisms of reward processing in both health and disease, including addiction and schizophrenia.

## 2. Materials and Methods

**Figure 1:**
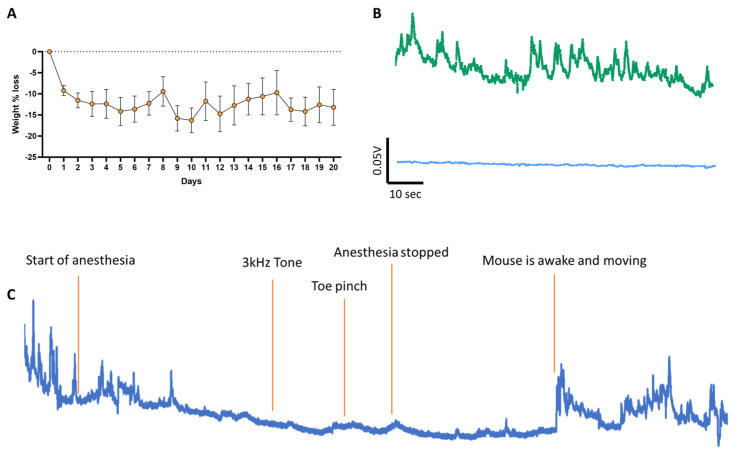
(**A**) Average mouse weight before and during training in the operant conditioning task. On day 0, mice are weighed, and their home cage water is replaced with 2% citric acid water. On days 0, 1, and 2, mice are handled to habituate them to the operator. Following this habituation period, mice undergo the three phases of the operant conditioning task (days 3 to 20). Mice exhibit a slight (~10%) weight loss due to reduced water intake, but their weight remains stable for the rest of the experiment, indicating that daily sucrose consumption during the task does not affect their weight. (**B**) Raw sample fluorescence traces recorded during baseline with excitation at 405 nm (bottom trace, blue), GCaMP6f isosbestic point, in comparison to calcium signal at 465 nm (top, green). (**C**) Sample trace of a trained mouse under anesthesia. The playing of the tone while the mouse is under anesthesia did not provide any direct sensory-related response, nor did a toe pinch, suggesting that dSPNs in the VS are not responsive to sensory stimulation.

**Animals.** Wild-type and transgenic mice expressing Cre-recombinase in D1-expressing SPNs (D1-cre) on a C57BL/6 background were used for these experiments. All animal procedures were approved by the Institutional Animal Care and Use Committee of the New York State Psychiatric Institute. Experiments were performed on 3- to 6-month-old male and female mice. No significant differences were observed between males and females; therefore, the data from both sexes were combined for analysis and presentation. C57BL/6 mice from Jackson Laboratory (Jax #000664) were used as control animals. D1-Cre transgenic mice were obtained from MMRC/Jax (ey262 D1-cre tg-drdla-cre) and crossed with C57BL/6 mice. Animals were kept on a reversed 12:12 light cycle (lights are off from 1100 to 2300 h) with free access to food and water. Behavior testing occurred during the daytime (between 1200 and 1600 h) and under red light to ensure minimal disturbance of the animals’ sleep cycle. On average, animals were housed in groups of 2 or 3 per cage. Mice were handled daily for the 3 days prior to experiments to habituate them to the operator. Each animal’s baseline weight was recorded on the first day of handling, while they had free access to plain water in their home cage. We then switched their home-cage water to water in which 2% citric acid was dissolved [14]. Mice were kept on a 2% citric acid water regime for the remaining of the handling period and during testing. We recorded their daily weight until the end of the experiment, when mice went back to the plain water regime (Figure 1A).

**Viral expression of GCaMP6f and fiber implants.** To achieve neuronal subtype-specific expression of the genetically encoded calcium sensor GCaMP6f in direct pathway SPNs, AAV vectors containing Cre-inducible GCaMP6f (AAV.9.Syn.Flex.GCaMP6f.WPRE.SV40, Addgene; TIter ≥ 2.1 × 13 GC/mL) were injected into the right VS by stereotaxic surgery, as shown previously [15,16]. During the surgery, a small skull craniotomy (1 mm × 1 mm) above the injection site was opened with a dental drill. A glass pipette attached to a Nanoject II (Drummond Scientific, Broomall, PA, USA) was filled with the GCaMP6f AAV and lowered to the target location (tip coordinates from bregma: AP +1.5 mm, ML −1.5, DV −4.3 mm). A total volume of 200 nL AAV vector was injected over 10 min. The pipette was left in place for 5 more minutes before removal. A 4 mm long 300 µm diameter optic fiber (Doric Lenses; MFC 200/245-0.37_4 mm_MF2.5_FLT) was subsequently lowered to the same coordinates. The skull was then covered with dental acrylic to secure the optic fiber in place. Animals were allowed to recover, and photometry experiments were performed 4 weeks after surgery, for optimal viral expression. Three to five days before experiments, animals were placed in an open field chamber to record baseline calcium signals and habituate to the tethered optical fiber. One control mouse was anesthetized to confirm the detection of calcium signals under baseline conditions. As expected, anesthesia abolished dSPN activity.

**Figure 2:**
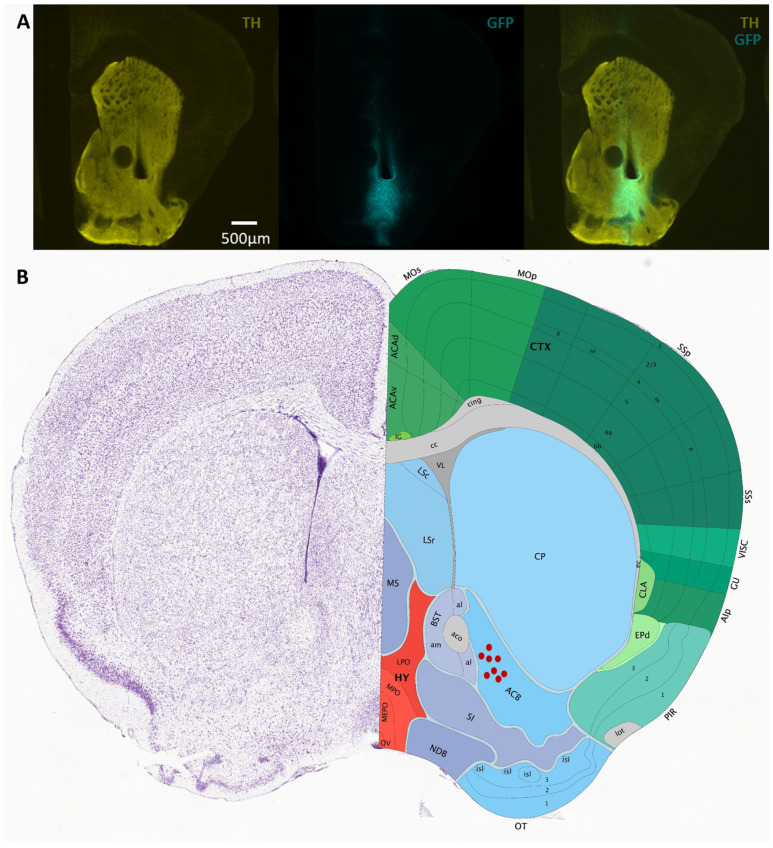
(**A**) Sample micrographs of coronal sections of the VS of D1-cre animals injected with AAV vectors containing Flex-GCaMP6f. GCaMP6f expression is confirmed by GFP immunolabeling (cyan) co-stained with TH antibody (yellow) to visualize the striatum. (**B**) Schematic of location of optic fibers for mice implanted in the VS (figure from Allen Brain Atlas).

**Photometry recording.** Calcium signals were recorded using a fiber photometry apparatus (Doric Lenses), as in previous work [15,16]. The system consisted of a console connected to a computer, a four-channel programmable LED driver, and two LEDs at 405 and 465 nm connected to fluorescence dichroic mini cubes and photometric multipliers tubes (PMTs). The 405 nm wavelength is the isosbestic point for GCaMP, where fluorescence does not change depending on cytosolic calcium concentration (Figure 2). Detection at this wavelength was used to remove background noise (movement artifact or GCaMP auto-fluorescence). The 465 nm excitation provides detection of GCaMP signal, where the intensity of fluorescence is proportional to cytosolic calcium concentration. Calcium signals from one cohort of animals were recorded for 5 min as the mice explored their home cage prior to treadmill testing. This is used as a baseline to evaluate whether there is any loss of signal over the several days of testing, as well as to habituate the animal to the optical cable (Figure 1B).

**Immunohistochemistry.** After the completion of the behavioral experiments, mice were terminally anesthetized (euthasol 240 mg/kg, 200 µL, i.p.) and intracardially perfused with PBS then 4% paraformaldehyde (PFA). Brains were extracted and post-fixed overnight (4% PFA, 4 °C). Coronal slices (100 µm) were obtained by vibratome (Leica VT 1200). Sections were rinsed with 0.6% Triton-X in 1× PBS (PBST; 6 × 40 min) and blocked in 10% normal donkey serum (NDS) in PBST (60 min, RT). Primary antibodies, chicken polyclonal GFP (ab13970 Abcam, Cambridge, MA, USA; 1:500) and rabbit polyclonal anti-tyrosine hydroxylase (TH; ab152 Abcam; 1:500), were applied in 2% NDS in PBST (48 h 4 °C) prior to washing (6 × 40 min PBST) and secondary incubation with species-specific Alexafluor IgG secondary antibodies (60 min RT, Invitrogen, Waltham, MA, USA; 1:500). Tissues were washed again in 0.1% PBST (6 × 40 min), then mounted using DAPI Fluoromount-G^®^ (0100-20, SouthernBiotech, Birmingham, AL, USA). Images were acquired using a 4.5× oil objective on an Olympus microscope (see sample images in Figure 2).

**Figure 3:**
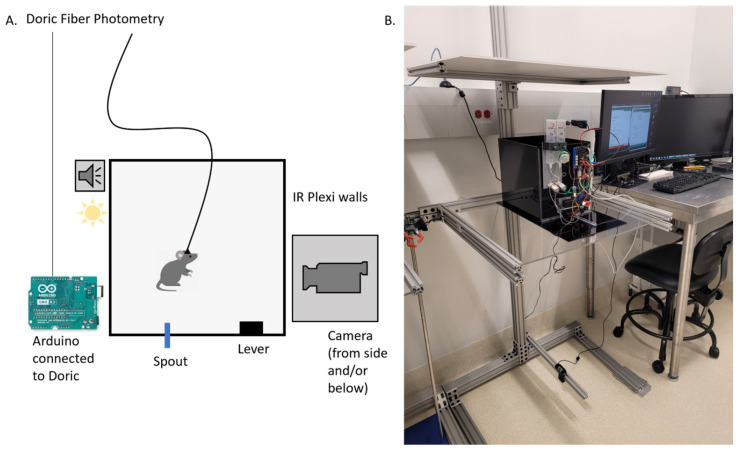
Schematic of the experimental setup. (**A**) Mice were placed in a rectangular box made of infrared-transmitting acrylic. An Arduino controlled various aspects of the experiment, including spout availability, reward delivery, lever presses, the force required to activate the lever, tone and light cues, and initiation of calcium imaging via a Doric Fiber Photometry system. (**B**) A camera was positioned below the box and another to the side, providing simultaneous views from both the bottom and side of the setup.

**Custom-built operant box.** To precisely control the animal’s behavior during instrumental learning, we designed an Arduino-controlled operant box (Figure 3). The enclosure was constructed using black infrared transmitting acrylic (ePlastic) to provide video recording in the dark with infrared cameras and infrared lights, with one camera positioned at the bottom and one on the side. Animals were trained to interact with a lever attached to a mini load cell (Sparkfun TAL 211) to measure the force applied to each lever press and a liquid reward spout attached to a rotating servo motor (Hitec HS-311) to control the insertion and removal of the spout. An Arduino microcontroller controlled all behavioral parameters via an open-source interface board (OM1, openmaze.org), including (a) the movement of the spout motor, (b) the lever sensor, (c) auditory cue duration and frequency, (d) visual cue LED color and intensity, (e) a synchronization signal to the fiber photometry system, and (f) initiation of the calcium recording, as well as providing information about the number and timing of licks and the amount of sucrose solution delivered with each lick.

**Operant conditioning task.** Mice undergoing experimental procedures were weighed prior to testing. All tests were performed in the afternoon between 1:30 pm and 4:30 pm, during the animals’ awake phase. All animals were assessed at 4–6 months of age. Mice were trained in a multi-phase operant task designed to assess reward–cue association. The task consisted of Phase I, a classical conditioning task in which mice were exposed to an auditory conditioned stimulus (CS) (3000 Hz, 60–80 dB, 5 s) paired with a reward (20% sucrose solution); Phase II, an operant task where lever presses were reinforced with a reward; and Phase III, a cued operant phase where lever presses were only rewarded during the presentation of the CS (Figure 1). The task was conducted in operant chambers equipped with sound generators, levers, and reward dispensers as described above (Figure 3). Mice were tested twice daily, with each test consisting of 5 to 10 trials on average, depending on the learning rate of each mouse. The learning rate was assessed by the experimenter based on whether the animal successfully interacted with the spout at least once during the test. Most animals reached this criterion within 5 to 10 trials per test. Behavioral responses, including lever presses and reward retrievals, were recorded using custom Arduino scripts integrated with the hardware and quantified into observable metrics using custom-built Python code. Across all phases, we computed the latency to the first lick (defined as the time it takes for the mouse to attempt the first lick) and the total number of licks per trial, and for Phase II and Phase III, we also computed the latency to first lever press and a total number of lever presses per trial. The process consisted of (1) parsing the Arduino data to identify each lick and/or lever press event within each trial, (2) computing the number of events per trial and the time from the beginning of the trial to the first event, and (3) identifying licks and lever presses that lead to rewarding (i.e., successful) or non-rewarding (i.e., unsuccessful) attempts. We classified a trial as Rewarded if there was at least one rewarded lick and Non-Rewarded if there were no rewarded licks. Comparisons between conditions (e.g., Rewarded vs. Non-Rewarded trials) were conducted using paired t-tests or ANOVAs, with significance set at *p* < 0.05. The Arduino and Python source codes are freely available on https://github.com/DSulzerLab/Operant_Phase_Training_Analysis (accessed on 1 December 2024).

**Calcium signal analysis.** Calcium signals were processed using Doric Neuroscience software (version 6.5.0) and custom-built Python code to remove background noise and detect individual calcium peak events, as shown previously [16]. The process consisted of (1) normalization of the data and removal of background noise, (2) synchronization of the calcium data with the corresponding Arduino outputs for each trial, and (3) identification and quantification of licks and lever presses and corresponding peak events (events amplitude). In detail, 465 and 405 signals were normalized using the formula F-ΔF/ΔF, where ΔF is calculated by a rolling mean of the overall trace. The 405 signal was subtracted from the 465 signal to remove movement artifacts and background noise. The resulting normalized trace is then analyzed using the Python custom-made code, which identifies individual peak events that have a peak amplitude greater than 2 standard deviations, at the time of the individual instance of interest (lick or lever press). Individual traces were inspected to ensure the accuracy of peak identification. Corresponding calcium traces were averaged to calculate the likelihood of a calcium peak event occurring at the same time as a given behavioral event. All data are presented as averages across multiple trials and multiple animals. The source code is freely available on https://github.com/DSulzerLab/Operant_Phase_Training_Analysis (accessed on 1 December 2024).

**Video analysis.** Behavioral experiments were recorded using a high-speed USB webcam (Sony PS3eye) and commercial video acquisition software (Kinovea-2023.1.2), synchronized to the Arduino/OpenMaze behavior system with an infrared LED, and analyzed post hoc using DeepLabCut [16,17] to track movement of individual body parts (the four paws, head, rear of the body, and tail were labeled as shown in Figure 1 and the Supplementary Materials Movie S1). Data were processed using Google Colaboratory, and approximately 30,000–40,000 iterations were sufficient for good-quality tracking. A sample of an analyzed video is shown (see Supplementary Materials Movie S1). The DeepLabCut results were processed with custom-built Python code to determine the distance to the spout and the distance to the lever, which were then correlated with calcium activity. The source code is freely available on https://github.com/DSulzerLab/Operant_Phase_Training_Analysis (accessed on 1 December 2024).

**Code accessibility and statistics and data reporting.** The code described in the paper is freely available online at https://github.com/DSulzerLab/Operant_Phase_Training_Analysis (accessed on 1 December 2024). The code is available as Extended Data. Data are presented throughout as mean ± SEM, where n is the number of animals. Comparisons were conducted by t-Test or 1- or 2-way ANOVA with appropriate post hoc tests detailed in the text, using Prism 9.0 (GraphPad, San Diego, CA, USA).

## 3. Results

### 3.1. Mice Show Rapid Learning During the Phase I Operant Conditioning Paradigm

To explore the behavioral responses to cue and action association with reward, we developed a customized operant conditioning paradigm. After 4 weeks of viral GCaMP6f expression and habituation to the operator, to promote learning but avoid water restriction, mice were put on a 2% citric acid water regime three days prior to testing and for the duration of the test [14]. Mice presented a slight weight loss (~10%) observed due to reduced water consumption, but within acceptable limits to prevent harm to the animals (Figure 1A; [18,19]). The weight then remained stable throughout the experiment, suggesting no impact on the mouse weight from daily sucrose intake.

**Figure 4:**
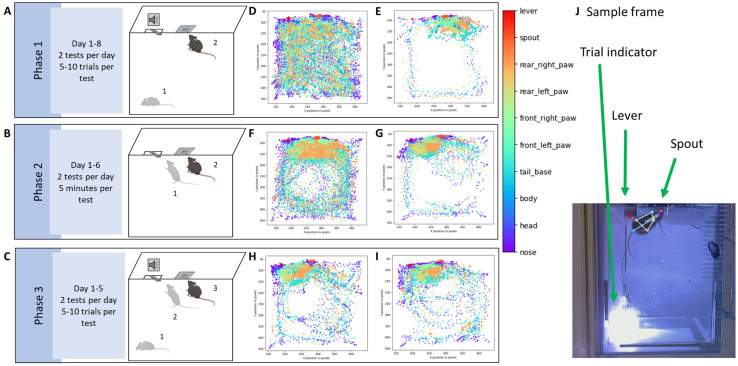
(**A**–**C**) Schematic of the behavioral protocol. (**A**) In *Phase I*, mice are placed in the operant box (step 1), where a spout (center of the wall at the top of the image) is always accessible. However, the spout delivers a sucrose reward only when the mouse licks it while a 3 kHz tone is playing (step 2). Mice learn to associate the tone with reward availability (classical conditioning). The tone lasts for 5 s, followed by a 15 s interval during which the spout does not deliver a reward, even when licked. This sequence repeats for multiple trials (each tone signals the start of a trial) daily, over 8 consecutive days. Two tests were performed each day. There were 5 to 10 trials in each test, depending on the success rate for each individual mouse, and to avoid satiation. (**B**) In *Phase II*, the tone is not played. The spout is only activated if the mouse presses a lever (operant conditioning; step 1). Each lever press initiates a trial, and the spout is released making the reward available (step 2), and this process continues for multiple trials daily over 6 consecutive days, with two tests each day. Within each test, trials were counted by each lever press event and lasted 10 s. Mice could press the lever as many times as they wished within the 5 min test. (**C**) In *Phase III*, the mouse is placed in the box (step 1) and the lever can only be pressed during the tone (step 2), and only then it releases the spout for the mouse to access the reward (step 3). The tone starts each trial, which repeats multiple times daily for 5 consecutive days. During this phase, there were two tests each day and 5 to 10 trials within each test. Behavior is recorded using an infrared camera placed beneath the box. DeepLabCut is used to track the positions of the mouse’s body parts, and an LED light signals the start of each trial (triggered by the tone in *Phases I* and *III*, or by the lever press in *Phase II*). (**D**–**I**) Schematic of DeepLabCut results. The legend on the right shows the color code for the different body parts. Each point represents the position of a body part in one frame, and all frames from a session are combined to create a 2D spatial representation. Samples from the same mouse across the three phases are shown. (**D**) Day 1 of *Phase I*: The mouse explored the entire box. (**E**) By day 8, the mouse spent most of its time near the spout, ignoring the rest of the box. (**F**) Day 1 of *Phase II*: The mouse was mostly near the spout but, since the spout only appears after pressing the lever, the mouse continued to explore the box. (**G**) Once the mouse learned that pressing the lever releases the spout, it spent more time around the lever and spout. (**H**,**I**) In *Phase III*, the mouse has already associated both the lever and spout with the reward and spent most of its time around these two objects. (**J**) Labeled frame from one mouse. The arrows indicate the LED light, the lever, and the spout.

The custom-designed operant box is equipped with a spout motor, a lever press sensor, and a tone generator, all synchronized with the photometry system (Doric Lenses; as described in the Materials and Methods). Behavioral responses are monitored using a video feed synchronized with photometric data, as shown previously [16]. This design provides precise control over the experimental environment, ensuring high-quality behavioral and neural data (Figure 3 and Figure 4J).

**Figure 5:**
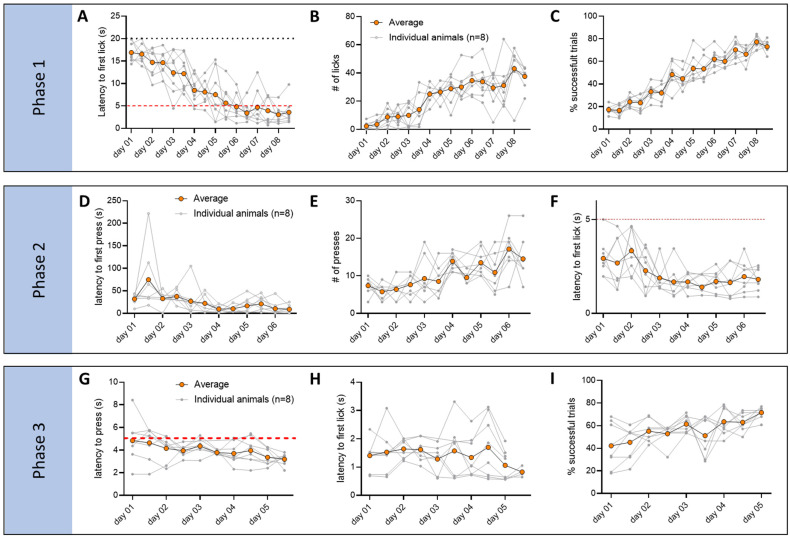
Behavior data of mice tested with our operant conditioning paradigm. For each graph, data are organized by day and test (x-axis, two tests per day, totaling 16 time points for *Phase I*, 12 for *Phase II*, and 10 for *Phase III*), and each data point is an average of all the trials within each test. The averages for each individual animal are shown in gray (n = 8), and orange indicates the average of all mice. During *Phase I*, mice learned to reach the spout more rapidly at the beginning of the tone ((**A**), latency to the first lick; RM 1-way ANOVA Day *p* < 0.0001, F_(3.768, 25.88)_ = 22.05, *Dunnett’s multiple comparisons test p*< 0.001 from day 4 to day 8) and make a higher number of licks ((**B**), # of licks; RM 1-way ANOVA Day *p* < 0.0001, F_(3.796, 26.57)_ = 19.02, *Dunnett’s multiple comparisons test p* < 0.001 from day 4 to day 8). Overall animals make more successful trials as training progresses ((**C**), % of successful trials; RM 1-way ANOVA Day *p* < 0.001, F_(3.153, 22.07)_ = 64.69, *Dunnett’s multiple comparisons test p* < 0.01 on day 3, *p* < 0.001 on day 4 to 8). During *Phase II*, mice learned to press the lever more rapidly ((**D**), latency to the first lick; RM 1-way ANOVA Day *p* < 0.01, F_(3.390, 24.96)_ = 6.262, *Dunnett’s multiple comparisons test p* < 0.01 from day 4 to day 6) and increase the total number of lever presses ((**E**), # of presses; RM 1-way ANOVA Day *p* < 0.05, F_(2.938, 21.90)_ = 3.697, *Dunnett’s multiple comparisons test* did not show any significance). There was a significant reduction in the time it took the animal to reach the spout after pressing the lever, demonstrating a learned association between the operant action and the reward consumption ((**F**), latency to the first lick; RM 1-way ANOVA Day *p* < 0.05, F_(3.399, 22.25)_ = 22.05, *Dunnett’s multiple comparisons test p* < 0.05 from day 4 to day 6). During *Phase III*, mice already exhibited a low latency to press the lever from the beginning of the tone and low latency to first lick after lever press ((**G**), latency to press, and (**H**), latency to first lick from lever press; both are not significant by RM 1-way ANOVA). There was however an increase in the number of successful trials, as indicated by the ratio of trials in which the mouse successfully pressed the lever during the tone and successfully consumed the reward after the lever press vs. the trials in which one or both steps were missing ((**I**), % of successful trials; RM 1-way ANOVA Day *p* < 0.05, F_(2.793, 19.55)_ = 4.978, *Dunnett’s multiple comparisons test p* < 0.05 on day 5).

The experiment followed a structured three-phase operant conditioning protocol, where freely moving mice associate an auditory cue and lever presses with sucrose rewards (Figure 4A–C). *Phase I* (Classical Conditioning) involved pairing a 3 kHz 5 s tone (conditioned stimulus, CS) with sucrose delivery and training the mice to associate the tone with the reward. Mice had access to a spout, but a reward was delivered only if the mouse licked the spout during the 5 s tone. This protocol was repeated for multiple trials daily over 8 consecutive days (Figure 4A). *Phase II* (Operant Conditioning) replaced the CS with a lever that, when pressed, made the spout available for 5 s, allowing the mouse to retrieve the sucrose. This phase lasted for 6 consecutive days with multiple trials per day (Figure 4B and Figure 5A). *Phase III* (Cued Operant Conditioning) combined the learned associations from *Phases I* and *II*. Mice were rewarded for pressing the lever only during the brief 5 s tone, merging the cue and motor elements of the previous phases. This phase lasted for 5 days (Figure 4C). We have found that 8 days for *Phase I*, 6 days for *Phase II*, and 5 days for *Phase III* were sufficient for the mice to reach stable behavioral responses (Figure 4C).

Body part placement showed significant changes in the box exploration after training. Comparison of body placement during the first day and last day of *Phase I* showed a clear switch of interest towards the spout, as indicated by a lack of exploration of the rest of the box (day 1 in Figure 4D and day 8 in Figure 4E). Exploration during *Phase II* showed an initial focus on the spout that then shifted to both the lever and spout (Figure 4F and day 6 in Figure 4G). During *Phase III*, there is no clear switch in exploration of the box, as mice are generally already focusing on the lever and spout from day 1 of that phase, and spend little time exploring the rest of the cage, which by this point is very familiar (Figure 4H and day 8 in Figure 4I).

Behavioral analysis during these phases (Figure 5) showed significant improvement and learning of the different steps of the experiment. In *Phase I*, mice showed a significant reduction in the time to reach the spout after tone onset (Figure 5A; RM 1-way ANOVA Day, *** *p* < 0.0001, F_(3.768, 25.88)_ = 22.05, *Dunnett’s multiple comparisons test* *** *p* < 0.001 from day 4 to day 8), and an increase in the number of licks (Figure 5B; RM 1-way ANOVA Day, *p* < 0.0001, F(3.796, 26.57) = 19.02, *Dunnett’s multiple comparisons test* *** *p* < 0.001 from day 4 to day 8) and a number of trials in which the animal successfully approaches the reward during the tone (Figure 5C, % of successful trials; RM 1-way ANOVA Day *** *p* < 0.001, F(3.153, 22.07) = 64.69, Dunnett’s multiple comparisons test ** *p*< 0.01 on day 3, *** *p*< 0.001 on day 4 to 8). In *Phase II*, the latency to press the lever significantly decreased (Figure 5D; RM 1-way ANOVA Day, ** *p* < 0.01, F_(3.390, 24.96)_ = 6.262, *Dunnett’s multiple comparisons test* ** *p* < 0.01 from day 4 to day 6), while the total number of lever presses increased (Figure 5E; RM 1-way ANOVA Day, ** *p* < 0.05, F_(2.938, 21.90)_ = 3.697, no significance found in post hoc tests). Importantly, after pressing the lever, there was a significant reduction in the time it took the mouse to reach the spout (Figure 5F; RM 1-way ANOVA Day, * *p* < 0.05, F_(3.399, 22.25)_ = 22.05, *Dunnett’s multiple comparisons test* * *p* < 0.05 from day 4 to day 6) in agreement with the DeepLabCut analysis, showing more time spent around the lever and the spout, indicating learned association between the action and the reward. In *Phase III*, mice exhibited a low latency to pressing the lever and to initiate licking, without any significant changes over the multiple days of testing (Figure 5G,H), while the rate of successful trials increased over the 5 days (Figure 5I; RM 1-way ANOVA Day, * *p* < 0.05, F_(2.793, 19.55)_ = 4.978, *Dunnett’s multiple comparisons test* * *p* < 0.05 on day 5), suggesting refinement of the skill.

### 3.2. Direct Pathway SPNs Process Motor Actions That Lead to Reward

**Figure 6:**
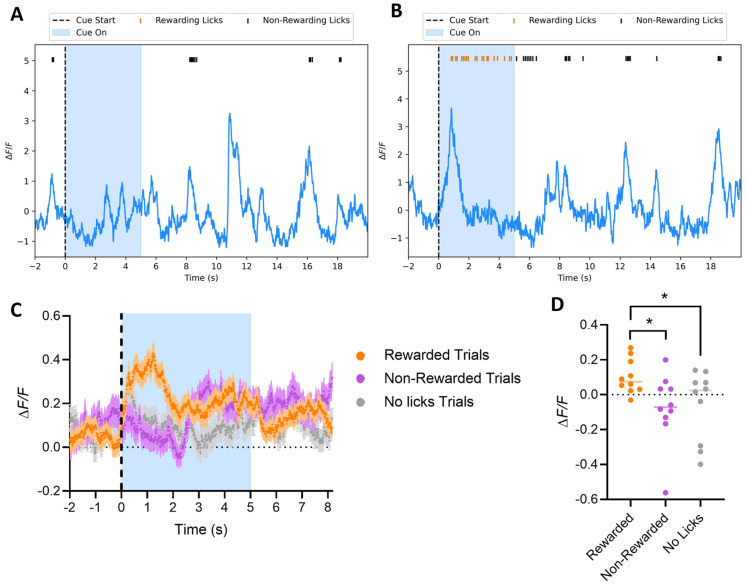
*Phase I*: Classical Conditioning. Mice have access to the spout, but the reward is only delivered if the mouse licks the spout during the tone. (**A**,**B**) Sample calcium traces of dSPNs in the VS from a *non-rewarded* trial (**A**) and a *rewarded* trial (**B**) show a peak in calcium activity at the start of each licking bout, followed by a decrease until the licking ends, with no significant difference in the amplitude of each licking bout action (whether leading to reward or no-reward). This suggests the activity is associated with the initiation of licking, rather than the cue itself (blue-shaded area). (**C**,**D**) Averaged calcium traces from dSPNs recorded during *Phase I*. Trials where the mouse successfully receives the reward are labeled as *rewarded* (orange), while those where the mouse licks outside the tone window and does not receive the reward are labeled as *non-rewarded* (magenta). Trials with no licks are labeled as *no licks* (grey). Calcium activity is significantly higher at the beginning of the tone during *rewarded* trials ((**A**), 2-way ANOVA Group *p* < 0.0001, *Tukey HSD post hoc test* * *p* < 0.05 for *rewarded* vs. *non-rewarded* and *rewarded* vs. *no licks*). The peak amplitude during the tone (max peak within the first 2 s of tone) was also significantly higher for *rewarded* trials ((**B**), 1-way ANOVA * *p* < 0.05, F_(1.892, 17.02)_ = 5.941, *Dunnett’s multiple comparisons test* * *p* < 0.05 for *rewarded* vs. *non-rewarded* and *rewarded* vs. *no licks*).

We next measured direct pathway neuronal activity in order to evaluate the requirement of their neural dynamics for the transduction of reward predictive cues into locomotion and reward acquisition. We used D1-cre mice expressing GCaMP6f to track dSPNs’ activity in the VS, explore their role during behavior, and more fully describe how this population of neurons encodes aspects of behavior during sensorimotor learning. Mice were implanted with an optic fiber in the same location and implant location, and expression of GCaMP6f was confirmed by immunohistochemistry (Figure 1B). An analysis of dSPNs’ calcium activity was carried out across the different phases of the experiment. Dopamine neurons that project to the VS respond to predictive auditory cues during learning [11]. Interestingly, in Phase I (Classical Conditioning), regardless of the day of testing, dSPNs did not show a strong response to the auditory cue itself. Instead, traces from non-rewarded (Figure 6A) and rewarded trials (Figure 6B) showed a peak in calcium activity at the beginning of each licking bout, followed by a gradual decrease, suggesting that the activity is more closely related to the initiation of licking rather than the auditory cue. The activity was significantly bigger in trials where the reward was successfully acquired (Figure 6C, 2-way ANOVA Group, **** *p* < 0.0001, Tukey HSD post hoc test * *p* < 0.05 for rewarded vs. non-rewarded and rewarded vs. no licks), suggesting that the association of the tone with the action was highly relevant for the activity of these neurons. The difference between the signals was confirmed by max peak amplitude between the groups of trials when were calculated within the first 2 s of the tone for each group (Figure 6D, 1-way ANOVA, * *p* < 0.05, F_(1.892, 17.02)_ = 5.941, Dunnett’s multiple comparisons test * *p* < 0.05 for rewarded vs. non-rewarded and rewarded vs. no licks). We conclude that dSPNs are active when the CS is heard and then a decision is made to lick, in contrast to a response to all perceived CS.

**Figure 7:**
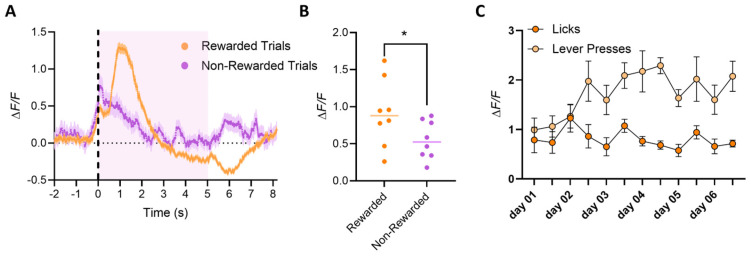
*Phase II*: Operant conditioning. Averaged dSPNs calcium traces recorded during *Phase II*. Mice must press the lever to make the spout available for reward consumption. Trials start at the pressing of the lever but are considered *rewarded* (orange) only if the mouse actively consumes the reward (licks are detected after the lever press); otherwise, the trial is considered *non-rewarded* (magenta). (**A**) Mice show a peak of calcium activity at the initiation of the lever press (dotted line; reward availability labeled with pink-shaded area), although peak amplitude is on average greater if the mouse moves to consume the reward (*rewarded* trials, orange) compared to trials where the mouse presses the lever but does not approach and lick the spout (*non-rewarded* trials, blue; 2-way ANOVA Group *p* < 0.0001, *Tukey HSD post hoc test* * *p* < 0.05 for *rewarded* vs. *no licks*). This suggests that dSPN neurons in the VS have higher activity when the mouse intends to press the lever and then lick the spout, compared to when the mouse accidentally presses the lever but does not intend to lick the spout. (**B**) Quantification of the peak amplitude at the maximum peak during the trial shows a much higher amplitude for trials, in which the reward is consumed versus trials that are not rewarded (2-tailed *t*-test * *p* < 0.05). (**C**) Average amplitude of peaks at the lever press (light orange) or at the first lick in a bout (dark orange), plotted by day of training. Interestingly, calcium peak amplitude increases over training, as mice become faster at pressing the lever and then reaching and consuming the reward (2-way ANOVA Group * *p* < 0.001, *Tukey HSD post hoc test* * *p* < 0.05, F_(1,14)_ = 24.21 for *presses* vs. *licks*, *Dunnett’s multiple comparisons test* * *p* < 0.05 for day and day 5).

In Phase II (Operant Conditioning), mice showed a peak in calcium activity at the initiation of the lever press (Figure 7A), regardless of whether the mouse moved to acquire the reward. However, trials in which mice intentionally pressed the lever to then acquire the reward showed a further increase in calcium activity, and this peak was on average greater in trials where the mouse consumed the reward versus trials where the mouse did not approach and lick the spout (Figure 7A; 2-way ANOVA Group, **** *p* < 0.0001, Tukey HSD post hoc test * *p* < 0.05 for rewarded vs. no licks). The quantification of peak amplitude further confirmed the higher activity in rewarded trials (Figure 7B; 2-tailed *t*-test, * *p* < 0.05). We conclude that dSPN neurons in the VS are more active when the mouse intends to press the lever and subsequently consume the reward than when the mouse presses the lever without intent to lick the spout (goal-directed behavior). This is consistent with the literature on the dopamine system and with the dSPN activity recorded in the dorsal portion of the striatum [20,21]. Interestingly, over the course of training, the average calcium peak amplitude increased as the mice became faster at pressing the lever and consuming the reward (Figure 7C; 2-way ANOVA Group, ** *p* < 0.001, Tukey HSD post hoc test * *p* < 0.05, F_(1, 14)_ = 24.21 for presses vs. licks, Dunnett’s multiple comparisons test * *p* < 0.05 for day 4 and day 5), indicating a learning effect on dSPN activity related to the goal-directed action, i.e., the action necessary to acquire the reward but not to the reward consumption itself.

To confirm that dSPNs in the VS are not directly responsive to sensory stimulation, we recorded calcium activity in a fully trained mouse under anesthesia. As expected, anesthesia completely eliminated all peaks of calcium activity, confirming that we are recording calcium signals from dSPNs (Figure 1C). We then played the tone but observed no response to the auditory stimulation. To further test whether dSPNs detect broader sensory inputs, we pinched the mouse’s toe and again saw no change in activity. Calcium activity only resumed when the anesthesia was stopped, and the mouse woke up and initiated movement. Together, these results indicate that dSPNs in the VS do not respond to the auditory cue in isolation, suggesting that sensory processing may be mediated by other regions. This suggests that while dopamine plays a significant role in reward prediction and cue associations, dSPNs may be more involved in the actual execution of rewarded actions rather than responding to predictive cues.

### 3.3. dSPNs’ Activity Remains Stable Around Goal-Directed Actions Once the Skill Is Acquired

**Figure 8:**
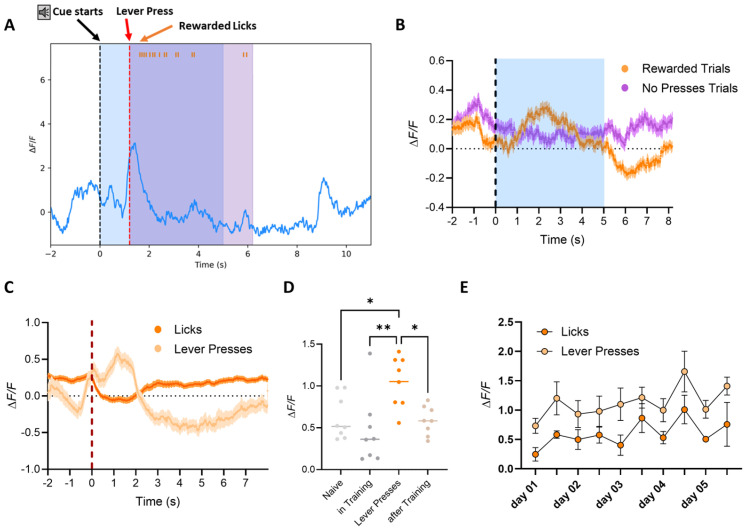
*Phase III*: Cued-Operant Conditioning. Averaged dSPN calcium traces recorded during *Phase III*, where mice were required to press the lever during the tone to make the spout available for reward consumption. (**A**) Analyzed sample trace of dSPN calcium signals recorded in the VS of a mouse performing *Phase III* of the operant conditioning task. The image indicates the start of the auditory cue (black dotted line), the initiation of the lever press (red dotted line), showing the increase in activity of the motor action (as explained in Figure 4), and the beginning of the lick event (each lick is labeled by an orange tick at the top of the image). Calcium activity is reduced for the duration of the lick event and only returns once the mouse is finished licking (when the spout retracts after the end of the reward availability period −5 s from the lever press). (**B**) Trials began at the tone onset and were classified as *rewarded* (orange) if the mouse pressed the lever within the tone’s time window and consumed the reward (licks detected after the lever press): Otherwise, the trial was categorized as a *no-presses* (magenta) trial. Mice displayed a peak in calcium activity when the lever was pressed within the time in which the tone was playing (blue-shaded area), with calcium signals showing a depression during reward consumption, similar to the activity observed in *Phase II*. The dotted line indicates the onset of the tone, but no specific tone response was detected during *Phase III*. (**C**) When signals were aligned by event onset, the data showed a significant difference between the peak at the initiation of the lever press (light orange) and the activity during reward consumption (dark orange; 2-way ANOVA Group, *p* < 0.0001, *Tukey HSD post hoc test* * *p* < 0.05 for *lever presses* vs. *licks*). (**D**) Calcium signals were recorded in the mouse home cage before training to detect baseline and acclimatize the animal to the optic fiber. Signals were then recorded after the mouse had completed the three phases of the experiment. Average peak amplitudes of both naïve (light grey) and trained animals (light brown) were comparable to signals detected during training (in-training values detected from day 3 of *Phase II*), indicating that there is no loss in signal after multiple days of recording. Nevertheless, the average peak amplitudes were higher for the peaks at lever press (1-way ANOVA Group, ** *p* < 0.01, F_(3,28)_ = 5.784, *Tukey HSD post hoc test* * *p* < 0.05 and ** *p* < 0.01 as indicated in the graph). (**E**) The average amplitude of calcium peaks during lever presses or at the beginning of the first lick bout, plotted by day of training, revealed that the calcium peak amplitude during lever presses (light orange) or the activity at lick events (light orange) did not increase over time. This result suggests that there was no significant learning progression in *Phase III*, as the animals were already proficient in the task from day 1 of *Phase III* and did not require an additional 4 days to improve their performance.

**Figure 9:**
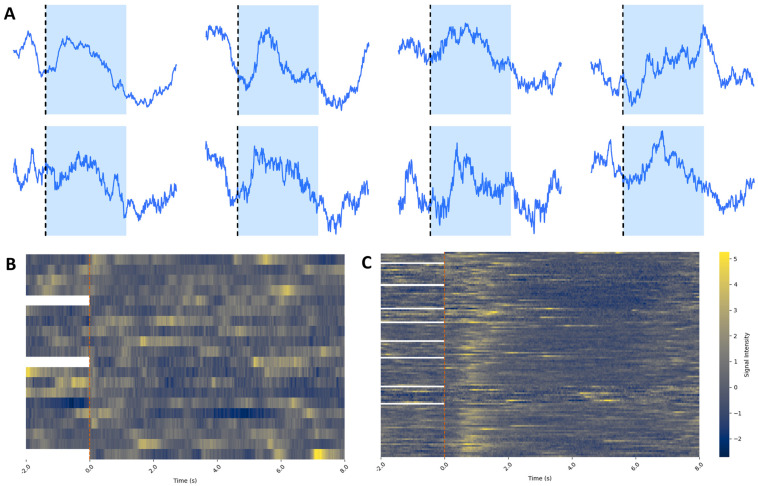
(**A**) Analyzed average sample traces of dSPN calcium signals during *rewarded* trials from each individual mouse recorded during *Phase III* of the operant conditioning task. The image indicates the start of the auditory cue (black dotted line and blue-shaded area), showing a consistent increase in activity (as explained in Figure 8). Each mouse peak timing was different, probably due to differences in the latency to approach the lever from the beginning of the tone and engagement with the lever. Nevertheless, all mice showed a peak in activity after the tone when they pressed the lever. (**B**,**C**) All calcium traces from *non-rewarded* (**B**) and *rewarded* (**C**) trials from one mouse during *Phase III*. Trials are aligned at time 0 (beginning of the trial) and plotted with the last 2 s of the previous trial. For the first trial of each test each day, there is no previous trial, so there is no data from the 2 s prior to the beginning of the trial. Yellow indicates high calcium activity, while blue indicates low calcium activity. (**B**) Data show no clear alignment of activity around the beginning of the tone (red dotted line). (**C**) There is a clear peak in activity immediately after the start of the tone, indicating when the mouse is pressing the lever. There is also a consistent depression in activity after the lever press, probably indicating the depression during reward consumption, consistent with the averaged traces in Figure 8.

In Phase III, dSPN calcium traces were recorded as mice were required to press the lever during a 5 s CS tone to make the spout available for reward consumption. Trials were classified as rewarded only if the mouse pressed the lever within the tone window and consumed the reward. Trials without lever presses were categorized as no presses trials. Averaged calcium traces showed a pronounced peak in dSPN activity at the moment of lever press, as observed in Phase II (Figure 8A,B). Similarly, the signal showed a depression during reward consumption, and no specific response to the tone was detected. dSPNs’ activity was primarily tied to the motor action (lever press) rather than the tone. When signals were aligned by event onset, a significant difference was observed between calcium peaks at the initiation of the lever press and the subsequent activity during reward consumption (Figure 8C; 2-way ANOVA Group, **** *p* < 0.0001, Tukey HSD post hoc test, *p* < 0.05 for lever presses vs. licks; also shown for a sample mouse in Figure 9), confirming that dSPNs are more active during the goal-directed action (lever press), as well to the initiation of the reward acquiring action (initiation of lick bout), indicating that calcium activity correlates with motor actions and licking events but not with the reward consumption per se (Figure 8 and Figure 9).

The average amplitude of calcium peaks during lever presses or at the onset of the first lick bout, plotted by day, showed no increase over time (Figure 8E), although it remained significantly higher than the overall average of peaks before, during, and after training (Figure 8D, 1-way ANOVA Group, ** *p* < 0.01, F_(3,28)_ = 5.784, Tukey HSD post hoc test * *p* < 0.05 for lever press vs. naive and lever press vs. after training, and ** *p* < 0.01 for lever press vs. during training). From behavioral data, we observed that mice were already proficient at the task from day 1 and showed no significant learning progression during the additional 4 days of training (Figure 5G,H). These findings suggest that once the task is learned, dSPN activity remains stable, reflecting proficiency rather than continued learning.

**Figure 10:**
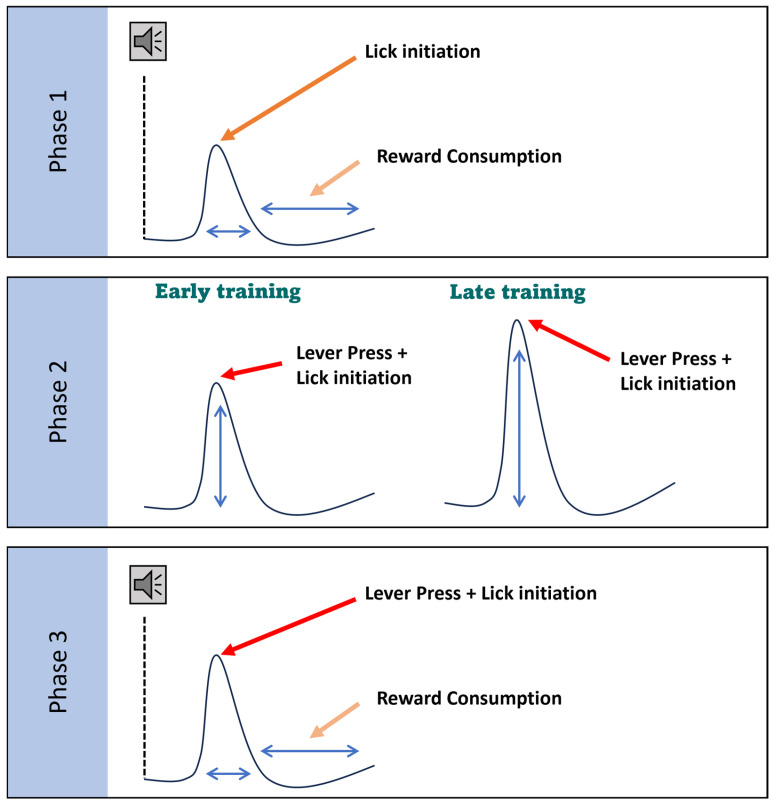
Working model. The schematic illustrates distinct components of dSPN activity associated with different goal-directed actions across learning phases, rather than changes in peak dynamics or kinetics, which can be variable depending on the timing of the actions. In *Phase I*, dSPNs show activity related to reward consumption following lick initiation. In *Phase II*, dSPNs are prominently active during lever pressing in late training, indicating their involvement in learned motor actions critical for reward acquisition. This activity pattern suggests that dSPNs are integral to reinforcing motor behaviors linked to obtaining rewards. By *Phase III*, after the task is learned, dSPNs display activity related to both lever pressing and reward consumption (wider peak), though the intensity of this activity is generally lower than the lever press activity during *Phase II*. This reduced activity implies that while dSPNs continue to support goal-directed actions, their role lessens as the behavior becomes more habitual. Overall, these findings indicate that dSPNs are essential for encoding the action–reward relationship during the initial learning stages, but their involvement remains constant once the task is habitual.

We conclude that VS direct pathway SPN activity peaks have multiple components, with a first component associated with the acquired skill and a second component that requires an action to acquire a reward, but not for the other motor components of the reward consumption. The activity of these neurons appears to be important for the motor action that leads to reward and not motor actions generally (Figure 10). Together, these results confirm that dSPN activity is linked to goal-directed behavior, particularly during trials where the mouse actively seeks and consumes a reward.

## 4. Discussion

Our findings suggest that the direct pathway in the VS plays a critical role in encoding the decision to engage in goal-directed actions that lead to reward acquisition, rather than processing sensory stimuli directly. Across the three phases of our operant conditioning task, dSPNs were consistently active only when a learned action was (a) initiated within the framework of a CS and (b) followed by reward consumption. In both awake and anesthetized animals, no response to the learned CS tone alone (the sensory cue) was detected, indicating that sensory processing of the CS may be handled by other regions, such as the tail of the striatum, which has been implicated in sensory integration.

Our data show that dSPNs do not merely reflect dopamine’s influence on activity or motivation but rather encode a decision-making process associated with the execution of operant behaviors. In contrast, dopamine neurons shift their activity from reward presentation to the associated cue as animals learn [11,12]. The release of dopamine in the striatum is known to enhance dSPN activity through D1 receptors [22,23], a step thought to enhance the likelihood of action initiation [7,24,25]. While our findings are consistent with these models, they further reveal that VS dSPNs are specifically responsive to actions that fulfill the learned CS–reward association. Thus, VS dSPN activity indicates a decision to perform a conditioned response (*lever press*) if it is to be followed by an unconditioned response (*licking*).

Detailed examination of the experimental phases further clarifies the conditions under which dSPNs are activated. During *Phase II*, where lever pressing was essential for reward acquisition, dSPNs exhibited high activity at the initiation of the lever press. This observation aligns with the role of the direct “go” pathway in promoting actions that lead to rewards [7,9,16]. By *Phase III*, when the task was well-learned, dSPN activity was still present but reduced, suggesting that these neurons are more engaged in the learning phase than in habitual execution. This refinement of dSPN activity mirrors findings from studies of motor learning, such as our recent work on treadmill running, where dSPN activity decreased as mice became more adept at the task [26]. The lack of a response to the tone alone in both awake and anesthetized states suggests that sensory processing of the CS may be managed by other striatal regions, such as the tail, and movement itself is coordinated by the dorsal striatum, while the VS is specialized for integrating the decision to perform a learned behavior within a CS–reward framework, encoding this goal-directed action rather than simply responding to sensory or reward cues in isolation.

The protocol we introduce here provides a means to study conditioned reinforcement, a mechanism in which a CS acquires the ability to reinforce drug-seeking behavior independently of the drug itself, which is thought to be a critical feature underlying relapse in addiction. These protocols may contribute to understanding real-world scenarios in which environmental cues, such as locations or paraphernalia, trigger drug-seeking behaviors long after the cessation of drug use [27].

In future experiments, it will be crucial to assess how dSPNs respond to more complex adaptive learning tasks that involve shifting behavioral rules. For example, testing with multiple cues that lead to different types of reward or aversive outcomes will elucidate how dSPNs support flexible decision-making in the context of changing contingencies. Additionally, tasks requiring discrimination between multiple reward sources, such as choosing a correct spout, would further characterize the role of dSPNs in goal-directed behavior. These types of paradigms are highly relevant for understanding decision-making in conditions including addiction or schizophrenia, which feature an inability to inhibit inappropriate responses along with impairments of auditory discrimination. Further research could parse how dSPNs contribute to response inhibition and how their activity may differ when engaging with multiple competing behavioral responses: Such insights would be further relevant to addiction and schizophrenia, where disrupted learning and maladaptive response patterns emerge. Finally, future research will be required to delineate the roles of dSPNs in different striatal regions during learning.

In summary, our experimental design, which combines operant conditioning, fiber photometry, and advanced behavioral analysis, has allowed us to precisely measure how dSPNs respond to the components of learned actions. We propose that dSPNs in the VS are crucial for initiating goal-directed actions within a learned framework of cue and reward, particularly during the acquisition phase of skill learning. Notably, we found that these neurons show heightened activity during the decision-making phase of the lever press but exhibit depression in activity during reward consumption, regardless of the reward’s presence, distinguishing their function from traditional dopamine neuron models. The finding that the VS supports operant decision-making processes rather than processing sensory stimuli or directly encoding reward has implications for understanding maladaptive behaviors, such as addiction and mental illness, where reward processing may be disrupted, and could refine our understanding of the striatum’s role in reinforcement learning and its implications for neuropsychiatric conditions.

## Data Availability

The raw data supporting the conclusions of this article will be made available by the authors on request.

## Supplementary Materials

The following supporting information can be downloaded at https://www.mdpi.com/article/10.3390/biomedicines12122755/s1, Video S1: Movie S1. Sample video from a mouse during Phase III, showing the animal pressing the lever during the CS (indicated by the LED infrared light in the top left corner of the video) to retrieve the spout and consume the reward. Video at 2x speed.

## Abbreviations

The following abbreviations are used in this manuscript:

VS	Ventral striatum
CS	Conditioned stimulus
dSPNs	Direct pathway spiny projection neurons
iSPNs	Indirect pathway spiny projection neurons
VTA	Ventral tegmental area
